# PheMaDB: A solution for storage, retrieval, and analysis of high throughput phenotype data

**DOI:** 10.1186/1471-2105-12-109

**Published:** 2011-04-20

**Authors:** Wenling E Chang, Keri Sarver, Brandon W Higgs, Timothy D Read, Nichole ME Nolan, Carol E Chapman, Kimberly A Bishop-Lilly, Shanmuga Sozhamannan

**Affiliations:** 1 Biotechnology, The MITRE Corporation, McLean, VA, USA; 2 Innovative Information Engineering and Biometrics, The MITRE Corporation, McLean, VA, USA; 3 Naval Medical Research Center, Biological Defense Research Directorate, Silver Spring, MD, USA and Henry M. Jackson Foundation, Rockville, MD, USA; 4 Department of Medicine, Division of Infectious Disease and Department of Human Genetics, Emory University School of Medicine, Atlanta, GA, USA

## Abstract

**Background:**

OmniLog™ phenotype microarrays (PMs) have the capability to measure and compare the growth responses of biological samples upon exposure to hundreds of growth conditions such as different metabolites and antibiotics over a time course of hours to days. In order to manage the large amount of data produced from the OmniLog™ instrument, PheMaDB (Phenotype Microarray DataBase), a web-based relational database, was designed. PheMaDB enables efficient storage, retrieval and rapid analysis of the OmniLog™ PM data.

**Description:**

PheMaDB allows the user to quickly identify records of interest for data analysis by filtering with a hierarchical ordering of Project, Strain, Phenotype, Replicate, and Temperature. PheMaDB then provides various statistical analysis options to identify specific growth pattern characteristics of the experimental strains, such as: outlier analysis, negative controls analysis (signal/background calibration), bar plots, pearson's correlation matrix, growth curve profile search, *k*-means clustering, and a heat map plot. This web-based database management system allows for both easy data sharing among multiple users and robust tools to phenotype organisms of interest.

**Conclusions:**

PheMaDB is an open source system standardized for OmniLog™ PM data. PheMaDB could facilitate the banking and sharing of phenotype data. The source code is available for download at http://phemadb.sourceforge.net.

## Background

High-throughput phenotype analysis, running multiple viral, bacterial, or eukaryotic strains through miniaturized assays, has long been used in the biotechnology industry and is becoming increasingly important in academic research as laboratory automation costs continue to decrease. With the advent of next generation genome sequencing instruments [1], allowing even small laboratories to sequence potentially hundreds of bacterial strains and viruses per year, very rapid functional profiling of a great number of samples becomes increasingly important.

One popular approach for high-throughput phenotype analysis is the OmniLog™ platform (Biolog Inc, Heyward, CA), an instrument designed primarily for metabolic and antibiotic resistance assays of bacterial and eukaryotic strains in 96-well microtiter plates. The instrument makes use of specially designed 'phenotype microarray' (PM) plate assays. For a standard bacterial metabolite assay, 20 plates 20 plates (1,920 wells) are used. In each well there is a different substrate (metabolite, antibiotic, etc) as well as a dye. Bacteria are deposited in each of the 1,920 wells and incubated in the temperature-controlled instrument. Fifty plates can be analyzed by the instrument at a time, and every 15 minutes for a user-defined number of days, a robotic camera takes a snapshot of each well and the instrument monitors the change in optical density of each well as the bacteria either grows, does not grow, or exhibits inhibition by the various compounds present in each well. In this way, the OmniLog™ can produce 4,800 data points every 15 minutes over the course of several days. A recent example of the use of the OmniLog™ system is the comparative metabolic analysis of two completely sequenced *Bacillus cereus *strains - ATCC 10987 and ATCC 14579. This analysis revealed differences in utilization of carbohydrates, peptides, amino acids and ammonia between strains that revealed potential adaptations to food borne pathogenesis [2].

To better facilitate the numerous challenges in managing the data produced in these experiments, which include processing data from raw form to interpretable summaries, raw strain and metadata storage, logical querying capabilities, statistical calculations, and representing results in publication quality figures, we have designed a software system called Phenotype Microarray DataBase (PheMaDB). This open source database and analysis system is designed to be used as a web server, facilitating the sharing of data and enhancing the user tools supplied with the OmniLog™ instrument.

## Construction and Content

### Implementation

The infrastructure for PheMaDB was created using the open source tools PHP http://www.php.net for the graphical user interface (GUI), R http://www.r-project.org for the analytical modules [3], MySQL http://www.mysql.com for the database, and Perl http://www.perl.org for parsing and uploading the OmniLog™ PM data into the database.

### Workflow

User accounts with associated access levels can be created within the system to allow the appropriate investigators permission for uploading, downloading, querying, or analyzing data. Once the user has been granted access to PheMaDB, the user follows a standardized process flow to input data and associate it with the following tables: Project, Species, Strain, Phenotype, and Genotype. URLs for specific projects are generated by a CGI script, allowing a means of direct linking of the PheMaDB to other databases. Upon linking to an entry in PheMaDB directly via an URL, the user is prompted for login information before being allowed to proceed.

The Kinetic table stores the time point data for each of the phenotypes in the OmniLog™ screen. Data from the OmniLog™ instrument is exported as comma separated values (.csv format) for upload into the system. Upon loading, the user links the kinetic dataset to a specific strain entry and a set of phenotypes.

For search and analytical functionality, the initial web pages present a choice of optional views. The user can select the search criteria of interest, then drill-down following a selection hierarchy of Project, Strain, Phenotype, Replicate, and Temperature for choosing records to analyze. The user can also choose to average all phenotype replicates for the same strain. The averaged result file can serve as an input into all the analysis modules except for the heat map plot, since this module has its own functionality to handle the replicate information.

**Figure 1:**
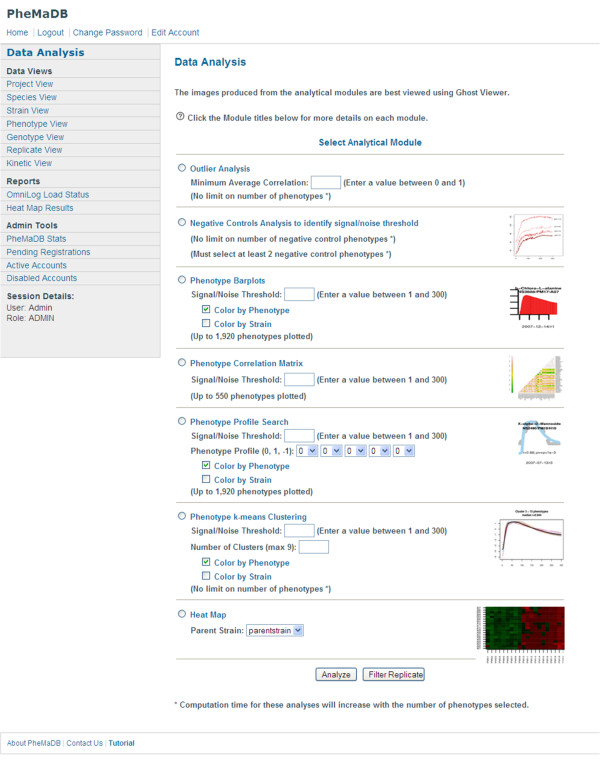
**PheMaDB analytical module selection page**. The analysis module menu page allows user to select specific analyses after identifying strains and phenotypes of interest.

Once the records have been generated, the analysis modules are presented to the user for selection (Figure 1). The following outline describes each of the seven modules:

#### 1. Outlier analysis

Allows users to identify aberrant phenotypes or strains within the batch of those selected. For each strain, phenotype and plate, the well and replicate combination values are interpolated to create vectors of equal and the median correlation for each group is calculated. Those records with a median correlation less than the user selected value are written out in an excel file.

#### 2. Negative controls analysis

Requires a set of negative control wells to identify the inherent noise threshold in the assay; non-negative controls are optional. Quantile curves are calculated for all negative controls selected (and non-negative controls, if provided) across the time points and the quantile curves are plotted, along with various threshold values that can be used to guide the signal/noise boundary determination.

#### 3. Bar plots

Graphs bar plots for all of the strain and phenotype records selected by the user, with shading specific to phenotype, strain, or both and outputs the images in a single file.

#### 4. Correlation matrix

Identifies similarities and/or differences between phenotype or strain profiles. This module calculates the pair-wise Pearson correlation between all strain, phenotype, plate, well, and replicate combinations selected. The values are interpolated to have equal lengths before correlation calculations. The correlation values are then output in an excel file, while an intensity plot is also output.

#### 5. Profile search

Allows users to specify a phenotype profile pattern over time and those records selected are compared against the user-defined profile for similarity. The values are interpolated to have equal lengths and both correlation coefficients and p-values are provided in each plot. The output includes a single image file with plots for each phenotype containing the user-defined profile pattern and the actual pattern for a phenotype. Profile shading is specific to phenotype, strain, or both.

#### 6. K-means clustering

Provides clustering of the records selected using a *k*-means algorithm. The values are interpolated to have equal lengths and the phenotypes are clustered into *k *user-defined clusters. The individual phenotypes are then z-score scaled and plotted for each cluster, as well as the centroid for the cluster with shading specific to phenotype, strain, or both. Both an image file and an excel file with cluster memberships and correlation values (to the cluster centroid for each cluster) are output.

#### 7. Heat map plot

Averages replicates for each strain and for each phenotype, computes the ratio of one or many test strain(s) to a user-selected parent strain. A Welch's modified t-test between each parent-test strain combination for all phenotypes is also calculated. Both an image file with an intensity heat map and an excel file is output with the ratios and p-values for each phenotype for all pair-wise parent-test strain contrasts.

All visuals are created in postscript (ps) format to provide high-resolution images based on vector graphics for zoom capabilities and publication-worthy figures.

## Results and discussion

### System Evaluation

In order to test the system and evaluate the ability of the analytical modules to identify meaningful patterns, we compared the phenotype profiles of a *Bacillus anthracis *mutant to its wild type parent. *B. anthracis *Sterne strain 7702 is sensitive to infection by a phage called AP50c and spontaneous AP50c resistant mutants were found to deposit an extracellular material on the bacterial surface [4]. We mapped the relevant mutations to the *csaB *gene and constructed a targeted *csaB *deletion mutant that was resistant to infection and also produced the extracellular material (Bishop-Lilly et al, manuscript in preparation). We hypothesized that the extracellular material may alter the permeability and entry of compounds and nutrients and thus might alter the phenotype profiles as measured by OmniLog™ phenotype microarrays.

For each experiment, forty 96-well plates were incubated (20 plates with wildtype and 20 plates with the csaB deletion mutant). Two replicates were performed for the wildtype AP50c strain and the csaB deletion mutant. There are 1,920 phenotypes in each set of 20 plates (20 plates × 96 wells). However, there are only a total of 1,200 distinct phenotypes since some phenotypes can be located in multiple wells on a plate per OmniLog's design.

**Figure 2:**
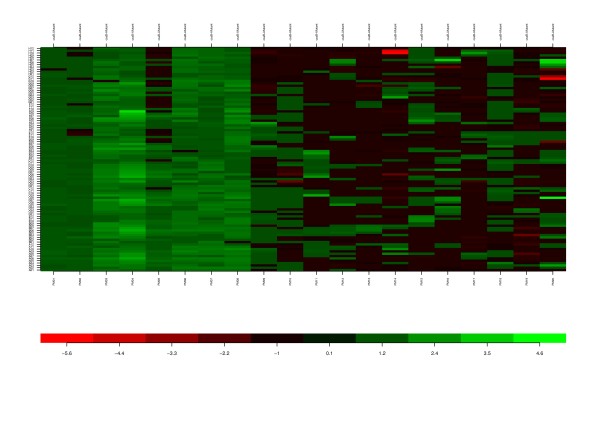
**Heat map of cell growth changes for AP50c mutant**. The heat map provides an overall picture of the cell growth impact on the 1,920 phenotypes for AP50c mutant strain as compared to the wild type strain. The green shaded areas (PM01-PM08) indicate better cell growth of mutant strain whereas the black and darkly shaded areas (PM09-PM20) indicate relatively similar cell growth of the mutant strain as compared to the wild type strain. The PM1 through PM20 data are presented as blocks of 20 columns. The rows represent the 96 wells in each of the PM plates (A01 through H12). The bar at the bottom provides visualization of the fold change between the mutant and the wild type parent strains.

We tested the hypothesis, and as shown in the heat map (Figure 2), a number of growth characteristics have been altered (albeit moderately) in the mutant as compared to the parent. Overall, there are a total of 48 compounds or nutrient treatments that were found to result in either positive or negative cellular growth in the mutant strain as compared to the wild type strain (p < 0.05; FC >= 2 or FC <= -2). The plates 1 through 10 contain growth metabolites such as nitrogen, carbon, and phosphorous sources. These columns are primarily shaded green, indicating growth promotion of the mutant strain.

In 40 of the 48 growth conditions, a significant increase in growth of the mutant strain as compared to the parent strain was observed. Of these 40 growth conditions, 31 were varied nitrogen sources. Among the 8 compounds and nutrients found to inhibit the growth of the mutant strain, one of the most effective compounds is 8-hydroxyquinoline (p = 9.97 × 10^-6^; FC = -5.39). This compound is a lipophilic iron-chelating agent that targets the anthrax toxin and inhibits *B. anthracis *growth in animals [5,6]. The mechanism of the enhanced growth inhibition of the *csaB *mutant in the presence of 8-hydroxyquinoline is not clear at this time. The complete list of cell growth results is provided in Additional file 1 (**Table S1**). The heat map module gives a snapshot of phenotype changes for this mutant strain as compared to the wild type parent strain for identification of growth differences in both magnitude and significance.

**Figure 3:**
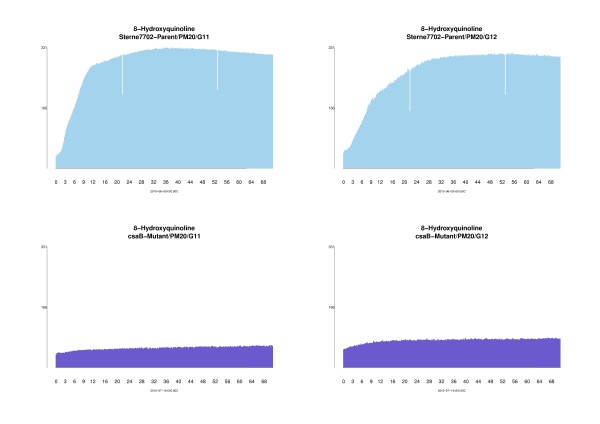
**Bar plots for 8-Hydroxyquinoline**. The bar plots indicate a decrease in cell growth for the AP50c mutant as compared to the wild type AP50c strain over a course of 72 hours. The top light blue bar plots represent 8-Hydroxyquinoline growth profiles located in PM20/G11 (left) and PM20/G12 (right) for the mutant strain. The bottom dark blue bar plots represent 8-Hydroxyquinoline growth profiles located in PM20/G11 (left) and PM20/G12 (right) for the parent strain.

To better illustrate the raw growth curves of the mutant and wild type strain over time, we used the bar plot module. The results from this analysis demonstrate the large magnitude of difference in the growth curves of the mutant and the wild type strain in medium containing 8-hydroxyquinoline (Figure 3). This plot also shows the variability between replicates (i.e. multiple wells that contain the same metabolite on the same plate) of the same strain and the peak times. These bar plots provide a quick and facile approach for visualization of phenotype differences between mutant and wild type *B. anthracis*, thereby demonstrating the utility and robustness of PheMaDB for this type of analysis.

### Future Work

Currently, basic quality control methods such as outlier analysis and negative control analysis are incorporated in the analysis modules. Outliers based on reproducibility between replicates can be tested. Background intensity ranges for allowing signal/noise thresholding can be determined. In the future, we plan to include additional quality control assessments such as detection of spatial biases.

PheMaDB was fully tested on Mac Operating Systems. Although most Linux/Unix Operating Systems should run PheMaDB, we plan to fully test PheMaDB in Linux/Unix platform to verify its compatibility.

While data storage for the OmniLog™ system has been the focus for initial development of PheMaDB, the database schema will support other types of phenotype data not necessarily produced by OmniLog™ (e.g. automated antibiotic sensitivity profiles) with appropriate loading scripts. Extension of the data types and implementation of new analysis modules will be the theme for future development of this open source project.

## Conclusions

PheMaDB is an open source web-based database management system that provides investigators with the ability to manage OmniLog™ data and strain metadata from the initial to terminal points of an experiment. It also allows users to share data easily via the web-based GUI and may serve to promote the public sharing of phenotype data. The system enables users to load and store the data, query across datasets, perform statistical analyses, and produce publication quality figures. PheMaDB provides storage and rapid determination of growth condition differences across a range of conditions and organisms.

## Availability and Requirements

The most recent release of PheMaDB source code is available at http://phemadb.sourceforge.net. The demonstration version of PheMaDB is available at http://binf.gmu.edu/wchang3/phemadb/pheno/index.php. The system is currently only supported on Mac operating systems. The README file included provides details on the installation as well as example files for test case analysis. PheMaDB uses GNU General Public License version 3 (GNU GPL3). The system requires the installations of PHP version 5.2 or higher, R version 2.0 or higher, apache version 2.2 or higher, Perl version 5.0 or higher, and MySQL version 5.0 or higher.

## Authors' contributions

BWH, KS, and WEC developed and implemented PheMaDB. BWH, KAB, SS, TDR, and WEC wrote the manuscript. NMEN and TDR provided data descriptions for the initial database design. KAB, TDR and SS provided the inputs for the analytical module designs. WEC tested all the features in PheMaDB and provided feedback for design improvements. CEC performed the OmniLog™ PM assays for the test case study. All authors read and approved the final manuscript.

## Supplementary Material

Additional file 1 **Table S1: Cell growth result**. For the worksheet designated, "All data", the columns A through D represent the location of each unique phenotype with the detailed descriptions of modes of actions. The columns E and F correspond to the fold changes and p-values, respectively. For the worksheet designated, "Filtered data", the phenotypes that have p-value < 0.05 and FC >= 2 or FC <= -2 are reported. Any phenotypes that have at least a 2-fold increase in cell growth for the ratio between the mutant strain and the parent strain are highlighted in yellow. All phenotypes that have at least a 2-fold decrease in cell growth for the ratio between the mutant strain and the parent strain are highlighted in pink. All the phenotypes that were in-between a 2-fold increase and decrease in cell growth were eliminated. Click here for file
